# Barriers to youth physical activity in urban green spaces: evidence from a Turkish city

**DOI:** 10.1186/s12889-026-26952-x

**Published:** 2026-03-10

**Authors:** İbrahim Pehlivan, Elif Karaca

**Affiliations:** 1 Department of Agricultural and Life Sciences, Çankırı Karatekin University, Çankırı, Türkiye; 2 Food and Agriculture Vocational School, Department of Park and Garden Plants, Çankırı Karatekin University, Çankırı, Türkiye

**Keywords:** Environmental barriers, Gender and age differences, Physical activity, Türkiye, Small-scale cities, Urban green spaces, Youth

## Abstract

**Background:**

Insufficient physical activity (PA) is a critical public health concern among young people in Türkiye. Despite the high prevalence of inactivity, limited research has focused on the 15–24 age group and the environmental factors influencing their PA behaviours, particularly in small-scale cities. This study aims to address this gap by examining PA behaviours, urban green space (UGS) use, satisfaction levels, perceived environmental barriers, and gender-specific differences among young people in Çankırı, within a study sample predominantly composed of female participants aged 15–19.

**Methods:**

This cross-sectional study was conducted with 400 students enrolled in public high schools and a university in Çankırı. A literature-based, original structured questionnaire was used as the data collection instrument. The questionnaire measured PA frequency, satisfaction with UGS, preferred types of UGS, and perceived environmental barriers. Data were analysed using descriptive statistics, ordinal and multinomial logistic regression analyses, as well as chi-square tests.

**Results:**

In this sample, only 28.5% of participants met the recommended PA level (≥ 3 days/week). Male participants in this sample were significantly more active than females (OR = 5.699, *p* = .003), and older participants (20–24) were more active than younger ones (15–19) (OR = 2.024, *p* = .002). Satisfaction with UGS positively predicted PA frequency (OR = 1.46, *p* = .001). Gender and age influenced UGS preferences, with older males favouring larger public parks. Although post‑correction statistical tests were non‑significant, descriptive patterns in this sample suggested gender‑related differences in perceived environmental barriers: male participants more often mentioned limited space, lack of sports fields, and stray animals, whereas female participants more frequently emphasised safety concerns.

**Conclusion:**

In this predominantly female and 15–19‑year‑old student sample, the findings suggest—exploratorily—that physical inactivity may be influenced by age, gender, environmental perceptions, and socio‑cultural factors, rather than individual choices alone. In particular, gender-specific perceptions of environmental barriers reveal that interventions aimed at promoting PA cannot succeed through a “uniform” approach. Therefore, the development of effective public health strategies at the local level necessitates holistic and context-sensitive planning approaches that recognise and respond to gender- and age-specific differences.

**Supplementary Information:**

The online version contains supplementary material available at 10.1186/s12889-026-26952-x.

## Background

In recent years, the rapid increase in urbanisation, technological advancements, and changing lifestyles have led to a decline in physical activity (PA) levels across society, particularly among the younger population [1–3]. In the absence of sufficient PA, it is projected that cardiovascular diseases, obesity, type 2 diabetes, and cancer will rise significantly by 2030 [4]. Similarly, global burden of disease studies identify physical inactivity as one of the major contributing factors to increased disease burden [5].

Located between Western Asia and Southeastern Europe and classified as an upper-middle-income country, Türkiye is increasingly facing a burden of chronic diseases, particularly from middle age onwards [6]. Türkiye ranks as the third most physically inactive country in Europe, with 44.4% of its population classified as physically inactive [7]. Furthermore, in terms of obesity prevalence—a condition closely linked to physical inactivity—Türkiye holds the highest position in Europe [8]. Additionally, the proportion of individuals aged 15 and above with insufficient PA levels in Türkiye is reported to be 42% [9].

Physical inactivity among young people is recognised as a significant public health concern. According to the World Health Organisation, more than 81% of individuals aged between 10 and 24 are physically inactive [10, 11]. Similarly, in Türkiye, sedentary lifestyles among the 15–24 age group have been increasingly prevalent, with the proportion of young people adopting a completely inactive lifestyle rising from 9.96% in 2019 to 16.34% in 2022 [12]. Adolescence is, in fact, a critical life stage during which PA behaviours are shaped. Globally, individuals aged 10–24 constitute approximately 24% of the world’s population, and health investments targeting this group are considered to offer triple benefits—immediate, during adulthood, and for future generations [13]. However, PA research predominantly focuses on early adolescents (aged 10–14) [13]. This indicates that the 15–24 age group is insufficiently addressed in PA studies and highlights the need to focus on this life stage. In this context, it is essential to understand the environmental, individual, and social determinants influencing PA behaviours among young people aged 15–24 in a multidimensional manner [14, 15]. The socio-ecological model developed by Bronfenbrenner [16] posits that individuals’ PA behaviours should be understood not only in relation to personal characteristics but also within the framework of their social relationships, environmental conditions, and broader contextual factors [17, 18]. According to the model, PA behaviours are shaped by a combination of personal, social, environmental, and policy-related factors.

In this context, gender emerges as a significant demographic variable influencing PA levels among young individuals [19, 20]. While the global gender gap in physical inactivity is approximately 5%, this disparity is considerably more pronounced in Türkiye, reaching nearly 20% [7]. This highlights the need for a more in-depth examination of gender-based differences within the Turkish context. Several studies conducted in Türkiye have shown that this inequality persists across different age and occupational groups. For instance, in a study conducted with university students, Dikmen et al. [21] reported that male students had significantly higher levels of total, vigorous, and moderate PA compared to female students. Similarly, Örs [22], in a study involving teachers, found that men exhibited higher PA levels than women. The literature emphasising gender-based inequality in PA also highlights that this disparity is not limited to differences in average PA levels. It underscores the need to consider the underlying social, motivational, and environmental factors contributing to this inequality [23]. Systematic reviews reveal that, in addition to individual-level factors, environmental elements such as the natural and built environment are also associated with PA, and they emphasise the importance of understanding this relationship [24, 25]. At this point, urban green spaces (UGS), with both their aesthetic and functional characteristics, are among the key environmental determinants influencing young people’s physical activity behaviours [14, 26–28]. Research indicates that greater availability of UGS is positively associated with PA levels among young people [29, 30]. In a systematic review including 14 studies, Mnich et al. [31] reported positive outcomes such as higher PA levels and lower sedentary behaviour among children and adolescents engaging in PA in open green spaces. Furthermore, several studies have shown that UGS are used by children, adolescents, and adults for PA and recreation, and may serve as important settings for youth PA in particular [32–34].

As noted by Orstad et al. [35], an individual’s PA behaviour is also influenced by their perceptions of the physical environment. Therefore, UGS play a determining role in PA behaviours not only through their mere presence but also through how they are perceived [36–39]. In this context, when considering the issues or opportunities that hinder or promote PA, a valuable approach to assessing environmental influences on young people’s perceptions is to identify the barriers to PA [25]. Barriers are defined as obstructive factors that prevent the execution of an activity and have a negative impact on the initiation and reinforcement of physical exercise habits [40]. Martínez Baena et al. [41] emphasise that gaining more insight into the barriers to PA behaviour may enable the implementation of more effective strategies to promote active participation in such activities. However, it should not be overlooked that perceptions of environmental barriers may differ by gender in the implementation of such strategies. In their study, Alejandre and Lynch [42] reported gender-based differences in the relationship between green space characteristics and children’s PA, noting that distance was a more significant contributor to inactivity among girls. Similarly, in a study by Veitch et al. [43], boys were more responsive to physical disorder, whereas girls were more sensitive to safety concerns and social restrictions. In a study conducted by Arumi-Prat et al. [44] with adolescents aged 16–18, distance was more frequently reported as a barrier by girls than by boys. In contrast to these findings, however, Portela-Pino et al. [45], in their study with adolescents aged 12–17, found no gender differences in the perception of environmental barriers. In Türkiye, studies focusing on environmental factors that hinder PA remain limited. In a study conducted in Akpınar and Cankurt [46] involving individuals aged 18–74, it was reported that women and younger individuals perceived environmental barriers more strongly than men and older adults. In another study by Akpınar [39] with adolescents aged 13–19 in Aydın, it was found that while the frequency and duration of green exercise among boys were not directly influenced by UGS characteristics, environmental factors—particularly distance—had a more pronounced effect on girls’ green exercise frequency and overall health status. Additionally, the presence of sports facilities was identified as a factor that increased PA among girls. Similarly, in a subsequent study with the same age group in Aydın, Akpınar [47] emphasised that environmental features posed different barriers for boys and girls, with safety and accessibility being more prominent concerns for girls.

Most studies have been conducted with adults and adolescents, and it remains unclear whether these findings are applicable to young people aged 15–24. Furthermore, while most research aiming to understand gender differences in PA behaviours among youth has been carried out in high-income countries, there is limited evidence from low- and middle-income countries [11, 48]. Considering the high rates of physical inactivity and pronounced gender inequality in Türkiye, this highlights the need for a more detailed and context-specific evaluation, as also emphasised by Tozduman and Gülle [7].

In this context, the present study was conducted in Çankırı, a small-scale province in Türkiye, with the aim of examining the PA behaviours of the young population at the local level. Due to its urbanisation dynamics, limited green space capacity, and socioeconomic diversity, Çankırı provides a suitable sample area for the local application of the socio-ecological model. This local focus expands existing research on the relationship between UGS and PA, which is predominantly conducted in large cities and high-income countries, and offers a new perspective for understanding the multi-level factors influencing youth participation in physical activity in upper-middle-income countries such as Türkiye. Accordingly, the study aims to contribute to the literature by examining the PA behaviours of students aged 15–24 in a study sample that primarily consisted of female adolescents aged 15–19, in relation to UGS use, environmental barriers, and gender‑specific differences. The hypotheses of the study are as follows:

- H1: The frequency of PA among young students differs significantly according to age, gender, and satisfaction with UGS.
- H2: The types of UGS preferred by young students for PA differ significantly according to age and gender.
- H3: The environmental barriers perceived by young students have a significant impact on participation in PA, and this impact varies by age and gender.

## Materials and methods

### Study area

**Fig. 1:**
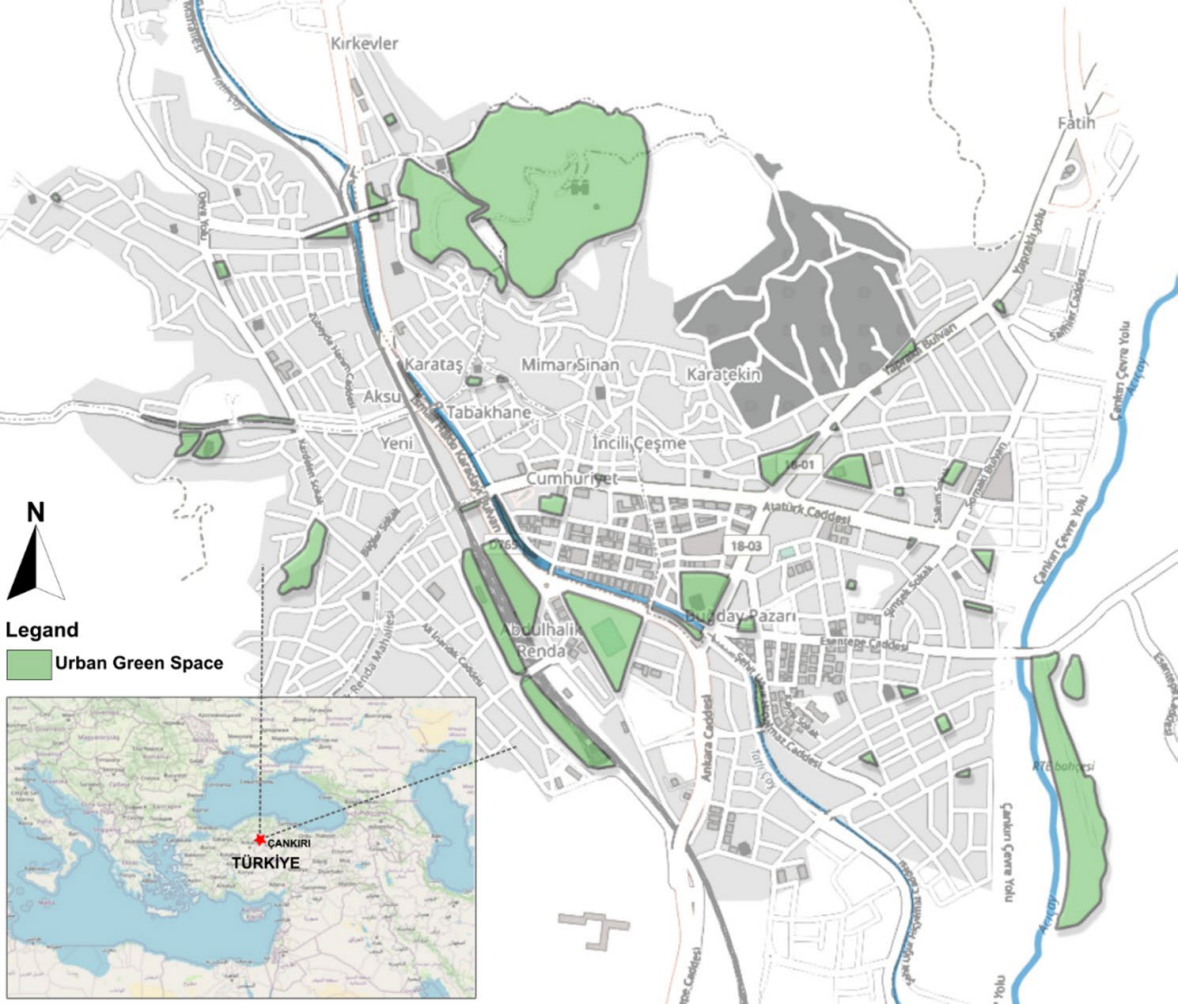
Study area. (The author drew this map by QGIS and Open Street Map)

This study was conducted in the Central district of Çankırı province, located in the north-western part of the Central Anatolia Region of Türkiye. The province is situated approximately 130 km northeast of the capital city, Ankara (Fig. 1). The area represents a typical urban setting within the context of Türkiye’s low- and middle-income cities; its physical infrastructure and sociodemographic structure are undergoing transformation due to migration from rural areas to the urban centre and rapidly changing urbanisation dynamics.

According to 2024 data, the total population of the Central district of Çankırı province is 89,142, with approximately 20.3% comprising young individuals aged between 15 and 24. Of this youth population, 9.7% are male and 10.6% are female [49].

World Health Organisation [50] recommends a minimum of 9 m² of green space per capita for a healthy urban living environment. This threshold is considered a fundamental lower limit in terms of both physical and mental health. However, a study conducted by Koçan and İbiş [51] determined that the amount of green space per capita in Çankırı province is only 4 m². This figure falls significantly below the minimum level recommended by the World Health Organisation.

The majority of green spaces in the city consist of neighbourhood parks, which serve at the neighbourhood scale and are smaller in size and more limited in terms of sports infrastructure compared to urban parks [52]. The Central district of Çankırı possesses a total of 65 UGS, which are significant for residents due to their physical and social functions and represent a potential resource for PA.

### Study sample

In this study, the sample was composed of students aged 15–24 residing in the Central district of Çankırı; however, the final sample predominantly consisted of female students aged 15–19. The research was conducted among students attending 13 public high schools and Çankırı Karatekin University, all located within the Central district. The only private high school within the provincial boundaries was not included in the study. The main reasons for this exclusion were that the student profile in public schools more broadly represents the youth of the region in terms of socio-demographic characteristics, access permissions were more readily obtainable, public high schools have a higher number of students, and fieldwork implementation was more sustainable in these settings.

Although the target population of the study comprised young individuals aged 15–24 residing in Çankırı province, it was not practically feasible to reach all individuals within this age group. Therefore, the data collection process focused exclusively on individuals with student status. This approach enabled the examination of a relatively homogeneous group in terms of age, educational level, and daily routines. Such homogeneity reduces the influence of external variables and enhances the reliability of the findings. Accordingly, participants were selected from among students attending all public high schools in the Central district of Çankırı and Çankırı Karatekin University, the only university in the province. As this selection was based on the deliberate identification of individuals appropriate to the research objectives, it is classified as purposive sampling. Within this defined student group, the sample was selected using the simple random sampling method.

According to 2022 data obtained from the Ministry of National Education and Çankırı Karatekin University, a total of 18,435 young individuals aged 15–24 were enrolled in educational institutions in Çankırı province [53, 54].

The sample size was calculated using Yamane’s formula (1), based on a 95% confidence level and a 5% margin of error; accordingly, a minimum of 392 participants was deemed sufficient. To enhance the reliability of statistical analyses and ensure generalisability, the sample size was increased. Students were invited to participate in the study through face-to-face interviews conducted in classroom settings at public high schools and the university campus. A total of 482 questionnaires were distributed, and 436 were returned. Of these, 400 were deemed valid, resulting in an effective response rate of 82.9%.

1 $$n=\frac{N}{{1+N(e}^{2})}$$

*n*: Sample size

*N*: Population size

e: Margin of error

### Questionnaire

The questionnaire used in this study was originally developed by Pehlivan [55] as part of a master’s thesis. The English version of the questionnaire is provided as supplementary material (Supplementary Material 1).

The questionnaire was developed following a comprehensive literature review examining the relationship between PA, youth, and UGS. Environmental and individual factors frequently emphasised in the literature were thematically classified, and original questionnaire items were constructed based on these themes. The items were not directly adapted from any standardised scale; however, they were structured with reference to valid and reliable studies in the relevant body of literature. During the questionnaire development process, the approach used by Wang et al. [36] to assess the frequency of green space use was adopted as a source of inspiration. Furthermore, to evaluate environmental barriers to participation in PA, Question 9 of the questionnaire was designed as a multiple-choice item, presenting 20 statements inspired by the works of Wang et al. [36], Akpınar [39], Akpınar [47] and Andersen et al. [56]. These items encompass both physical (e.g., lack of sports facilities, small size of the area) and perceptual (e.g., lack of perceived safety, presence of stray animals) barriers. The relevant question also includes an “Other” option, allowing participants to report barriers not previously defined. To enhance the content validity of the questionnaire, expert opinions were sought from three academics specialising in urban planning, landscape architecture, and statistics regarding the 20 barrier items and other questionnaire questions. The experts evaluated the items in terms of conceptual relevance and clarity for the target population; based on their feedback, revisions were made to certain items. Due to time constraints, a pilot study could not be conducted; however, expert evaluations provided a preliminary content check.

In this study, only selected items from the questionnaire were used in the analyses (Q1, Q2, Q4, Q5, Q7, Q9, and Q11). The majority of these items are single-item measures (e.g., gender, age, frequency of PA, level of satisfaction) and do not constitute a scale; therefore, a conventional internal consistency analysis (e.g., Cronbach’s Alpha) was not applied.

The items related to Question 9 of the questionnaire (e.g., Q9A) were analysed independently in the study, with each item subjected to separate chi-square tests. Therefore, internal consistency analyses (e.g., Cronbach’s Alpha) were not applied to these items either. Instead, item-level descriptive statistical analysis was conducted; the distribution of responses to single items (e.g., frequency, percentage, variance) was examined. Items showing clustering at extreme values were carefully evaluated in terms of measurement reliability. Additionally, the relationship between each item and demographic variables such as gender was assessed using chi-square tests, and significance levels (*p*-values), effect sizes (Cramer’s V), and confidence intervals were reported.

In this study, PA level was assessed based on participants’ self-reports and weekly frequency (days/week), in a manner similar to the structure of the Global PA Questionnaire (GPAQ) developed by the World Health Organisation. Participants were asked, “How many days per week do you engage in physical activity?” and responses were categorised as “daily”, “≥3 days per week”, “2 days per week”, “1 day per week”, “less frequently”, and “no PA”. This measure was analysed in comparison with the World Health Organisation’s recommended minimum level of weekly physical activity (≥ 3 days/week).

### Research design

This study was conducted using a cross-sectional research design, which involves a data collection approach aimed at describing the current state at a specific point in time. The data were collected once, through face-to-face survey administration, between October and November 2022. In the study, variables such as participants’ perceptions of UGS and their level of satisfaction with these areas were examined based on their current circumstances. This design aims to reveal existing relationships and variable distributions rather than investigating changes over time or causal relationships. Participation in the study was entirely voluntary, and all participants were provided with detailed information in advance regarding the purpose of the research, the use of the data, and confidentiality assurances. The research protocol was approved by the Ethics Committee for Social and Human Sciences at Çankırı Karatekin University, and written informed consent was obtained from all participants.

### Data analysis

The data obtained from this study were analysed using the SPSS 26.0 software (IBM Corp., Armonk, NY, USA). The analysis process was conducted in four main stages:

- Descriptive Statistics: The demographic characteristics and PA levels of the participants were summarised using frequency (n) and percentage (%) distributions.
- Ordinal Logistic Regression Analyses: Ordinal logistic regression analyses were conducted to evaluate the effects of demographic variables on PA frequency and satisfaction, as well as to determine the relationship between satisfaction and PA frequency. In the models, gender, age group, gender × age interaction, and UGS satisfaction were included as independent variables. Effect sizes were reported as odds ratios (OR) with 95% confidence intervals (CI).
- Multinomial Logistic Regression Analyses: Demographic variables predicting the types of UGS preferred by participants for PA were analysed using multinomial logistic regression.
- Analysis of Environmental Barriers: To assess the relationship between environmental factors hindering PA and gender and age groups, separate Chi-square (χ²) tests were conducted for each barrier item. The Bonferroni method was applied for multiple testing correction, with the adjusted significance threshold set at p < .0025. After correction, none of the associations reached statistical significance; therefore, chi-square results were treated as descriptive only, and no further inferential analyses were performed.

## Results

### Demographic characteristics and self-reported PA levels

The study sample consisted of 400 students aged 15 to 24, with a markedly imbalanced distribution. Specifically, the gender composition included 275 females (68.7%) and 125 males (31.3%). Regarding age, 283 participants (70.8%) were between 15 and 19 years old, whereas 117 participants (29.2%) were between 20 and 24 years old. (Table 1). These descriptive findings reflect the activity patterns of this specific sample and should not be generalized to all youth aged 15–24.

**Table 1:** Demographic characteristics

Characteristics	Category	*n*	%
Gender	Female	275	68.7
	Male	125	31.3
Age	15–19	283	70.8
	20–24	117	29.2

In this study sample, a critically low level of PA was observed. Among the participants, 50.3% (*n* = 201/400) engaged in PA less than once per week. Only 28.5% (*n* = 114) of all participants met the World Health Organisation’s recommended minimum threshold for weekly PA (i.e., 3 or more days per week) (Table 2).

**Table 2:** Physical activity frequencies

	How many days per week do you engage in physical activity?						
	Daily	≥ 3 days per week	2 days per week	1 day per week	Less frequent	No physical activity	Total
*n*	53	61	85	75	86	40	400
%	13.3%	15.3%	21.3%	18.8%	21.5%	10.0%	100.0%

## Effects of gender and age on PA levels

To examine the effect of gender and age variables on the frequency of PA, an ordinal logistic regression analysis was conducted. A three-step model was developed for this purpose. The first model included only the gender variable; the second model incorporated the age variable; and the third model included the gender × age interaction. All three models were found to be statistically significant (Table 3).

**Table 3:** Ordered logit models fit for PA frequency by gender, age, and interaction

					Overall Model Test		
Model	Deviance	AIC	BIC	*R*²_*N*_	χ²	df	*P*-value
Gender	1399	1411	1435	0.00750	7.99	1	0.005^*^
Age	1393	1407	1435	0.01274	13.60	2	0.001^*^
Gender X Age	1389	1405	1437	0.01664	17.78	3	< 0.001^*^

*R²N*  Nagelkerke *R*²

* *p* < .05

According to the results of the ordinal logistic regression analysis presented in Table 4, gender has a statistically significant effect on the frequency of PA. Male participants reported significantly higher levels of PA compared to female participants (β = 1.74, SE = 0.595, OR = 5.699, *p* = .003), although this pattern should be interpreted within the context of the sample’s female‑dominant composition. Similarly, age was found to have a significant effect, with participants aged 20–24 being twice as active as those aged 15–19 (β = 0.705, SE = 0.230, OR = 2.024, *p* = .002); however this pattern may partially reflect the overrepresentation of younger adolescents in the dataset. The interaction between gender and age was also statistically significant (β = −0.92, SE = 0.45, OR = 0.399, *p* = .041); however, given the limited and uneven subgroup sizes, this pattern should be considered exploratory. In this study sample, male participants reported higher levels of PA than females; however, this difference was less pronounced in the 20–24 age group, and the negative interaction coefficient may suggest that the increase in PA frequency with age is more limited among males compared to females. Nevertheless, the imbalance in group sizes limits the stability of this finding.

**Table 4:** Ordered logistic regression results predicting PA frequency by gender, age, and interaction

Variable	Category	Estimate (β)	SE	z-value	Odds ratio (OR)	95% CI	*P*-value
Gender	Male (Ref: Female)	1.740	0.595	2.92	5.699	[1.781,18.391]	0.003^*^
Age	20–24 (Ref: 15–19)	0.705	0.230	3.07	2.024	[1.292, 3.181]	0.002^*^
Gender X Age		-0.920	0.450	-2.04	0.399	[0.164, 0.962]	0.041^*^

*SE* Standard error, *Ref.* reference, *CI* Confidence Interval

* p < .05

### Effects of gender and age on preferred UGS types

To identify the types of UGS preferred by participants for PA, a multinomial logistic regression analysis was conducted. A three-step model was developed for this purpose. All three models were found to be statistically significant (Table 5).

**Table 5:** Multinomial logistic regression models fit for preferred UGS by gender, age, and interaction

					Overall Model Test		
Model	Deviance	AIC	BIC	*R*²_*N*_	χ²	df	*P*-value
Gender	858	866	882	0.0183	11.4	2	0.003^*^
Age	851	863	887	0.0292	18.2	4	0.001^*^
Gender X Age	842	858	890	0.0438	27.4	6	< 0.001^*^

*R²N*  Nagelkerke *R*²

* *p* < .05

The results of the multinomial logistic regression analysis presented in Table 6 indicated that age had a statistically significant effect on the likelihood of preferring large public parks. Specifically, individuals aged 20–24 in this study sample were less likely to prefer large public parks than those aged 15–19 (OR = 0.283, 95% CI [0.144, 0.557], *p* < .001). The interaction between gender and age also had a significant effect on the type of UGS preferred for PA (β = 1.765, OR = 5.843, 95% CI [1.583, 21.566], *p* = .008). In the study sample, this finding suggests that the effect of age on preference varies by gender. In particular, male participants aged 20–24 appeared, in this exploratory analysis, to be more likely to prefer large public parks compared to female participants aged 15–19. In our study sample, this may indicate that, as age increases, males tend to favour larger and better-equipped green spaces, whereas this preference remains more stable among females. Gender alone did not have a statistically significant effect on the preferred type of UGS (*p* > .05). However, given the skewed sample distribution, these findings should again be interpreted as sample‑specific rather than generalizable.

**Table 6:** Multinomial logit regression results predicting preferred UGS by gender, age, and interaction

Preferred UGS for Physical Activity	Variable	Category	Estimate (β)	SE	z-value	Odds ratio (OR)	95% CI	*P*-value
Nearby neighbourhood parks	Gender	Male (Ref: Female)	-0.353	0.944	-0.374	0.703	[0.1105, 4.470]	0.709
	Age	20–24 (Ref: 15–19)	-0.417	0.308	-1.353	0.659	[0.3602, 1.206]	0.176
	Gender X Age		0.417	0.724	0.576	1.518	[0.3675, 6.267]	0.564
Large public parks	Gender	Male (Ref: Female)	-1.418	0.872	-1.626	0.242	[0.0439, 1.338]	0.104
	Age	20–24 (Ref: 15–19)	-1.263	0.346	-3.654	0.283	[0.1436, 0.557]	< 0.001^*^
	Gender X Age		1.765	0.666	2.650	5.843	[1.5833, 21.566]	0.008^*^
(Ref: Residential green spaces)								

*SE* Standard error, *Ref.* reference, *CI* Confidence Interval

* *p* < 0.05

### Gender and age differences in satisfaction with UGS

To examine satisfaction levels with UGS used for PA, an ordinal logistic regression analysis was conducted. The gender variable did not have a statistically significant effect on satisfaction levels (OR = 2.909, *p* = .110). Similarly, neither the age variable (OR = 1.066, *p* = .802) nor the gender × age interaction (OR = 0.672, *p* = .417) were found to be statistically significant. These results indicate that, within this sample, participants’ satisfaction levels with UGS did not differ significantly across gender or age.

### Effect of UGS satisfaction on PA frequency

In this study sample, the ordinal logistic regression analysis revealed that participants’ satisfaction levels with UGS had a statistically significant effect on the frequency of PA. As shown in Table 7, the model was found to be significant (χ²(1) = 10.7, *p* = .001).

**Table 7:** Ordered logit model fit for PA frequency by UGS satisfaction

					Overall Model Test		
Model	Deviance	AIC	BIC	*R*²_*N*_	χ²	df	*P*-value
UGS Satisfaction (Q11)	1396	1408	1432	0.00999	10.7	1	0.001^*^

*R²N*  Nagelkerke *R*²

* *p* < .05

As shown in Table 8, each one-unit increase in satisfaction level was associated with a 1.46-fold increase in the likelihood of engaging in PA more frequently (OR = 1.46, 95% CI [1.16, 1.83], *p* = .001). In the study sample, this finding indicates that participants who are more satisfied with UGS tend to engage in PA more frequently.

**Table 8:** Ordered logit regression results predicting PA frequency by UGS satisfaction

Variable	Estimate (β)	SE	z-value	Odds ratio (OR)	95% CI	*P*-value
UGS Satisfaction (Q11)	0.375	0.116	3.25	1.46	[1.16, 1.83]	0.001^*^

*SE* Standard error, *Ref.* reference, *CI* Confidence Interval

* p < .05

### Environmental barriers to PA

In this study, chi-square analyses were conducted separately for each item to examine the relationships between gender and environmental factors that hinder PA (Table 9). Following Bonferroni correction, the threshold for statistical significance was set at *p* < .0025. Post-correction evaluations indicated that none of the inhibitory factors were statistically significant (*p* > .0025). Although these results were not statistically significant after correction, descriptive patterns within this sample indicated that male participants tended to report physical environment barriers (e.g., limited area size, lack of sports fields, presence of stray animals) more frequently, whereas female participants more often reported safety concerns. These patterns are sample-specific and should be interpreted cautiously given the overrepresentation of female adolescents. Observed patterns were interpreted in light of theoretical relevance and prior literature rather than statistical significance.

**Table 9:** Gender differences in environmental barriers to PA (pre-Bonferroni correction)

Barriers	Gender							
		No (n/%)	Yes (n/%)	χ^2^	*P*-value	df	Cramer’s V	95% CI
Limited area size	Female	227 (82.5%)	48 (17.5%)	4.26	0.039	1	0.103	[0.00102, 0.237]
	Male	92 (73.6%)	33 (26.4%)					
Lack of sports fields	Female	242 (88.0%)	33 (12.0%)	6.21	0.013	1	0.125	[0.0270, 0.297]
	Male	98 (78.4%)	27 (21.6%					
Lack of perceived safety	Female	227 (82.5%)	48 (17.5%)	4.16	0.041	1	0.102	[-0.245, -0.0194]
	Male	113 (90.4%)	12 (9.6%)					
Presence of stray animals	Female	266 (96.7%)	9 (3.3%)	6.92	0.009	1	0.131	[0.0567, 0.490]
	Male	113 (90.4%)	12 (9.6%)					

*CI* Confidence Interval, Bonferroni, *p* < 0.0025

To examine the relationships between age groups and environmental factors that hinder PA, chi-square analyses were conducted separately for each item. However, the results indicated no statistically significant associations between the inhibitory factors and age groups (*p* > .05).

## Discussion

### Prevalence of general physical inactivity

In this study sample, physical inactivity was highly prevalent, with a substantial proportion failing to meet the World Health Organization’s recommended daily levels of PA. This observation aligns with Öztürk [12] “Youth Health Report”, which reveals an increase in sedentary lifestyles among the 15–24 age group across Türkiye. Although this study sample shows patterns consistent with national data, these findings should be interpreted within the specific demographic composition of the sample.

At the macro level, Türkiye is classified as an “upper-middle-income” country and described as a “highly unequal middle-income country” [57, 58]. This socioeconomic context is closely linked to PA rates. Data from the Statistical Office of the European Communities [59] show that Türkiye ranks among the countries with the lowest leisure-time PA rates compared to European Union member states. Specifically, only 14% of young people aged 15–24 in Türkiye report engaging in PA at least once a week during their leisure time, whereas the EU average is 65% [59]. This situation is considered to be directly related to the macro-level defined in Bronfenbrenner [16] socio-ecological model and is largely attributed to socioeconomic inequality in Türkiye. Indeed, socioeconomic inequality is assumed to have a particularly adverse impact on leisure time PA, which typically require access to resources [43].

This inequality is also evident in Çankırı. Regional income distribution data indicate that 34.1% of households in the central district fall within the lower and lowest income groups, meaning one in three households faces significant socioeconomic disadvantage [60]. Similarly, Timur et al. [61] report that most students in Çankırı have very low incomes, restricting their ability to participate in desired activities. To mitigate these constraints, local governments should prioritise strategies such as organising free sports events and outdoor exercise programmes, and providing free or discounted access to sports facilities. These recommendations may be supported not only at the individual level but also at the environmental level. In particular, increasing the number of publicly accessible UGS for young people should be considered a key factor in promoting participation in PA.

In addition to socioeconomic inequalities, structural factors such as inadequate infrastructure, cultural habits, and social environment also constitute significant barriers to PA in Türkiye [62]. Limited facilities and financial constraints directly affect young people’s access to leisure activities. Comparative research by Yılmaz et al. [63] shows that Turkish youth report more limitations related to facilities and income than their peers in Germany, a disparity linked to differences in economic development. In Çankırı, the absence of integrated transport infrastructure and insufficient environmental arrangements—such as bicycle paths—further restrict opportunities for PA [64]. In this regard, increasing local government investments in bicycle lanes, walking trails, and green spaces accessible via public transport may be considered a strategy to promote young people’s participation in PA. Nevertheless, these interpretations reflect contextual reasoning and should not be generalized beyond the specific population included in this study.

### Relationship between PA frequency, gender, and age

As emphasised in Bronfenbrenner’s [16] socio-ecological model, at the micro level, an individual’s age and gender significantly influence PA behaviour. In this context, the international literature indicates that male individuals participate in physical activity at higher levels compared to females [65, 66]. In our study sample, the findings were consistent with this trend, and studies conducted within the context of Türkiye also present similar results [67, 68]. However, in a multi-country study conducted by Pengpid et al. [69], which included countries with varying levels of development, it was observed that PA levels among women in Türkiye were slightly higher than those of men. Nevertheless, Pengpid et al. [69] emphasised that in most previous studies conducted in Türkiye, men were found to be more physically active. In this predominantly female and 15–19 aged sample, this finding from Çankırı may be attributed to participants’ individual characteristics and behavioural preferences; however, it gains further significance when evaluated within the context of gender inequalities in Türkiye. Tüfekçi and Bulut [70] report that female students perceive more restrictions on participating in PA compared to their male counterparts, particularly due to factors such as social pressure, lack of motivation, and insufficient income. Similarly, Öztürk and Koca [71] emphasise that despite historical progress in women’s participation in leisure-time PA, gender-based constraints such as family pressure, safety concerns, and economic barriers remain strongly influential. Moreover, in countries with traditional social structures such as Türkiye, women’s participation in PA is also shaped by cultural factors including privacy, social norms, and gender roles [72]. In this context, the lower levels of PA observed among female participants in this study sample may not solely be explained by individual preferences, but rather by macro-level cultural norms, gender roles, and structural inequalities, as outlined in Bronfenbrenner’s [16] socio-ecological model. These effects may be more pronounced in cities like Çankırı, which have a more conservative social structure. This situation necessitates the design of intervention strategies that take into account multidimensional factors. Planning exercise spaces specifically for women, promoting peer-supported group activities, and establishing women-led sports groups are among the effective strategies that may enhance motivation [73]. In addition, awareness campaigns targeting families and social circles may help reduce social pressure on women and support their participation in PA. However, because females were substantially overrepresented in our study sample, the observed gender gap reflects sample‑specific dynamics and should not be interpreted as an indication of gender differences among all young people in the region.

Age is another important demographic variable influencing the frequency of PA. In our study sample, students in the 20–24 age group were found to participate in PA at higher levels compared to those in the 15–19 age group. This finding contradicts some international studies. For instance, van Sluijs et al. [13] and Mayo et al. [74] reported a decline in PA levels with increasing age. This discrepancy may be associated with the structure of Türkiye’s education system. In particular, students aged 15–19 are subject to intense academic pressure due to their preparation for university entrance examinations, which limits the time they can allocate to PA [75, 76]. This situation can be considered a structural barrier to young people’s participation in PA. In this context, strategies are needed to facilitate access to PA during examination periods. More effective implementation of physical education classes in schools, integration of short exercise programmes into the exam preparation process and increasing the availability of accessible PA spaces around schools may serve as effective interventions to reduce inactivity levels in this age group.

In our study sample, it was observed that the effect of age on PA frequency varied by gender. However, because subgroup sizes were limited and unbalanced, these interaction patterns should be interpreted as exploratory rather than conclusive. This exploratory nature is due to the limited statistical stability of the subgroup analyses. The descriptive patterns within this sample suggest that male students’ motivation to engage in PA tends to decline with age, whereas female students may maintain a more stable level of participation. This pattern was also confirmed in a study conducted by Mayo et al. [74] involving young people aged 15–24, which reported that the age-related decline in PA was more pronounced among males than females. The gender-specific variation in the effect of age highlights the need for intervention strategies to be designed with sensitivity to both age and gender. In particular, the decline in motivation among males as they grow older necessitates the development of targeted programmes for this group. Furthermore, considering that the rate of change in PA levels differs by gender, interventions should be adapted to address lifestyle changes at different ages for each gender.

### UGS use and barriers

As defined in Bronfenbrenner’s [16] socio-ecological model, the exosystem level encompasses physical and institutional environmental factors that do not involve direct interaction with the individual but exert indirect influence. In alignment with this level, our findings from this study sample indicate that PA behaviour is affected by the spatial characteristics of UGS as an environmental factor. According to the research results from this sample, although students in the 20–24 age group report higher levels of PA compared to those aged 15–19, they tend to use large public parks less frequently. While this may appear to contradict the expectation that more active individuals prefer better-equipped spaces, it can be explained by differences in activity type and spatial preference. Students aged 20–24 may engage in PA in more accessible locations, such as on-campus areas or through low-intensity activities like individual walking. However, some studies focusing on this age group have reported a preference for comprehensive landscapes offering dynamic sports and recreational facilities [77]. This discrepancy may be related not only to methodological differences but also to participants’ social habits and environmental opportunities. Indeed, the limited sports infrastructure in UGS in Çankırı may directly influence these preferences. Therefore, it is recommended that UGS planning consider not only demographic variables but also local socio-cultural habits and environmental resources.

In this study sample, our findings indicate that the effect of age on UGS preference varies by gender. Specifically, male students aged 20–24 are more likely to prefer large public parks compared to female students aged 15–19. As noted by Akpınar [47], this difference may be associated with women’s concerns regarding accessibility and safety. The literature frequently emphasises that women tend to prefer spaces that are closer and perceived as safer [78, 79]. Therefore, the fact that most large parks in Çankırı are located on the outskirts of the city may pose limitations for women in terms of both access and safety. This finding aligns with the descriptive patterns observed in our study. In the study sample, female participants more frequently reported a lack of safety compared to male participants, which is consistent with previous literature highlighting gender-specific safety concerns. Studies conducted in Türkiye [80, 81] and international literature [82, 83] have identified safety concerns as one of the most significant environmental factors limiting women’s participation in PA. In this context, gender-sensitive planning strategies should be developed to enhance women’s sense of safety and encourage their use of UGS. The literature indicates that design elements such as clear sightlines, adequate lighting [84, 85], and regular maintenance [86] positively influence women’s perceptions of safety. Therefore, UGS in Çankırı that are planned with these criteria in mind may positively affect women’s use of such areas. Furthermore, ensuring the active participation of women in planning and management processes may contribute to the development of spatial solutions that support gender equality.

Although gender-related differences in perceived barriers were not statistically significant after Bonferroni correction, the descriptive pattern in this study sample suggests that male participants tended to emphasise physical environment factors such as insufficient space, lack of sports facilities, and the presence of stray animals. This differentiation aligns with gender-specific PA preferences reported in the literature. Male individuals are noted to prefer larger and better-equipped areas, particularly for team sports such as basketball and football, whereas females tend to engage more in individual and low-intensity activities [87, 88]. In this context, the fact that parks in Çankırı are predominantly neighbourhood-scale and possess limited PA infrastructure may explain why male participants perceive environmental barriers such as “limited space” and “lack of sports fields” more strongly. Accordingly, expanding existing neighbourhood-scale parks or planning new, larger-scale green spaces with diversified infrastructure may provide suitable environments for the intensive and competitive PA preferred by young men.

A notable descriptive trend in our study sample was that male participants appeared to report the presence of stray animals as a barrier to PA more often than females. This observation contradicts the existing literature, which generally suggests that women are more sensitive to this issue [89, 90]. This discrepancy indicates that perceptions of environmental barriers may vary locally within the context of gender. In particular, the uncontrolled increase in the number of stray dogs in Türkiye has become a significant public health concern affecting participation in physical activity. Kırışık and Öztürk [91] state that stray animals constitute a serious environmental threat that affects not only PA but also daily life. In the specific context of Çankırı, the reasons why male participants perceive this issue as a more prominent barrier remain unclear. Therefore, it is recommended that qualitative research be conducted to better understand how environmental risk perceptions are shaped in relation to gender.

### Satisfaction with UGS

In this study sample, satisfaction with UGS had a significant effect on PA frequency. Participants who reported higher levels of satisfaction were observed to participate in PA more frequently. This finding is consistent with previous research. Gardsjord et al. [92] and Demirkan [93] demonstrated that green spaces designed to meet user needs increase satisfaction levels and promote regular PA. However, when evaluating this relationship, caution must be exercised regarding assumptions of causality. Physically more active individuals may use green spaces more frequently, which in turn may enhance their satisfaction with these areas. As emphasised by Sallis et al. [94], there are bidirectional interactions between the physical environment and PA. Therefore, while environmental features may influence PA, it is also possible that active individuals perceive and utilise environmental elements in different ways.

In this context, the design of UGS that can enhance young people’s satisfaction levels and promote participation in PA is of critical importance. It is considered essential for local governments not only to increase the quantity of green spaces but also to improve these areas in terms of accessibility, safety, diversity of facilities, and opportunities for social interaction. Furthermore, adopting participatory planning processes based on user feedback may render green spaces more responsive to the needs and expectations of young people, thereby increasing satisfaction levels.

On the other hand, our study found no statistically significant differences in satisfaction levels with UGS based on gender or age. This finding suggests that contrary to the demographic differences frequently emphasised in the literature, participants in this study sample exhibited similar perceptions of satisfaction. This may be explained by the fact that UGS in the city are predominantly neighbourhood-scale and possess similar facilities. Additionally, the scale used to measure satisfaction did not include subdimensions, which may have limited its sensitivity to demographic variables such as age and gender. In this context, future studies may benefit from assessing satisfaction through subcomponents such as “safety”, “facilities”, and “accessibility”, which could help reveal demographic differences more clearly.

As a result, in this study sample PA levels were low and appeared to be associated with multi-level factors—namely age, gender, satisfaction levels, and environmental barriers. Participants aged 20–24 were more physically active than those aged 15–19, who were under intense examination pressure, thereby highlighting a context‑specific structural barrier. Another key finding in this study sample was the gender-based differentiation in perceived environmental barriers. As expected, female participants identified “lack of safety” as the primary obstacle, whereas male participants more frequently reported “lack of sports fields” and the “presence of stray animals”—a factor less emphasised in the literature. Although non-significant, these descriptive patterns may tentatively point to the need for age‑ and gender‑sensitive planning strategies in Çankırı. In this context, it is recommended that local governments aim to increase the per capita amount of green space to meet the minimum standards proposed by the World Health Organisation. This includes expanding accessible, safe, and well-equipped green spaces around schools and university campuses. To enhance perceptions of safety among young girls, gender-sensitive design principles should be prioritised in planning. Examples from cities such as Stockholm, Yangon (Myanmar), and Barcelona, which have implemented planning strategies focused on young girls, may serve as useful references for local governments in Türkiye. For young men, the provision of specific sports facilities (e.g., basketball and football fields) is recommended. For the 15–19 age group, PA programmes integrated into academic calendars should be developed. Furthermore, local governments should address the issue of stray animals as a direct public health and PA barrier. In addition, planning processes involving local community participation—such as focus group discussions and individual interviews—may contribute to the development of solutions that directly reflect the needs of young people. Taken together, these findings from this predominantly female and 15–19‑year‑old sample highlight the need for multi‑level interventions to support PA participation among young people with similar demographic characteristics, particularly within local government and public health policy contexts.

### Limitations and future research

In this study, PA data were collected through self-reporting. This method may involve limitations such as recall bias and hypothetical responding. Similarly, the qualitative characteristics of UGS were assessed solely based on participants’ perceptions, which may hinder the objective determination of environmental influences. For future research, it is recommended that spatial indicators such as legibility, integration, and permeability be analysed using methods such as Geographic Information Systems (GIS) or space syntax, in order to systematically examine individuals’ modes of interaction with their environment. Furthermore, to measure behaviours such as PA and park usage more reliably, the use of technological tools such as accelerometers, GPS-based tracking systems, or mobile applications may enhance data validity and improve comparability across different contexts.

Satisfaction with UGS was also assessed based on participants’ self-reports. In the literature, validated standard instruments such as the NEWS/NEWS-Y (Neighbourhood Environment Walkability Scale – Youth version) and PANES (Physical Activity Neighbourhood Environment Survey) have been developed to measure environmental perception and spatial interaction dimensions in the context of PA. The absence of such tools represents a methodological limitation that restricts the construct validity of the study. Future research is advised to employ these types of scales to enable a more systematic and comparable assessment of environmental perception.

Although the content validity of the questionnaire items was ensured through expert qualitative evaluations, quantitative validity analyses such as the Content Validity Ratio (CVR) or Content Validity Index (CVI) were not conducted. This constitutes a methodological limitation in terms of the validity of the measurement instrument. In future studies, a more systematic assessment of content validity has the potential to enhance the reliability and comparability of research findings.

The cross-sectional design of the study allows for the examination of relationships between variables only at a single point in time, which limits the ability to draw causal inferences. To more robustly assess causal relationships, longitudinal approaches that track changes over time or intervention-based experimental designs may offer a stronger methodological framework.

The restriction of the study to the central district of Çankırı necessitates caution when generalising findings to young individuals living in cities with differing socio-economic structures. Although the sample consisting solely of individuals with student status offers advantages in terms of accessibility and homogeneity, it poses a risk of sampling bias due to its limited representation of the entire youth population aged 15–24. Including more diverse socio-demographic groups could contribute to a more comprehensive understanding of PA behaviours among young people.

In the study, 68.7% of participants were female and 70.8% were aged 15–19 years, which may limit the generalisability of gender- and age-based comparisons. Therefore, given the uneven and limited subgroup sizes (e.g., gender and age groups), the statistical stability and validity of subgroup and interaction analyses are compromised. Therefore, these findings should be interpreted strictly as exploratory rather than confirmatory. These imbalances likely resulted from the voluntary nature of participation. Moreover, due to the absence of population-level data required for weighted analyses, such methods could not be applied to the current dataset. Future research should aim to establish more balanced and representative samples in terms of both gender and age distribution to enhance the validity of comparative analyses.

In this study, only age and gender variables at the individual level were included in the analyses. Key individual health indicators that influence PA behaviour, such as body mass index (BMI) and general health status, were not assessed due to their absence in the dataset. Similarly, although the socioeconomic status of Turkey and Çankırı was addressed in general terms, individual-level socioeconomic variables such as income and parental education level were not available in the dataset. These omissions may have led to an incomplete representation of the individual-level components of the socio-ecological model. Incorporating such health and socioeconomic indicators into the data collection process in future research may contribute to a more holistic application of the model and a more comprehensive understanding of PA behaviour.

## Conclusion

This study offers a local‑level contribution to the literature, which is predominantly shaped by high‑income countries, by examining the PA behaviours of students aged 15–24 in Çankırı within a study sample predominantly composed of female participants aged 15–19, in the context of gender, age, and UGS utilisation. The findings from this specific sample suggest that physical inactivity cannot be explained solely by individual choices; rather, it is shaped through interactions with age, gender, environmental perceptions, and socio-cultural norms. In this study sample, gender‑specific perceptions of environmental barriers indicate that interventions aimed at promoting PA cannot succeed through a ‘uniform’ approach. Therefore, the development of effective public health strategies at the local level necessitates holistic and context-sensitive planning approaches that recognise and respond to gender- and age-specific differences. In this context, the planning of UGS as a health strategy may be regarded as a significant societal investment, offering ‘triple benefits’—for the present, for adulthood, and for future generations.

## Supplementary Information

Supplementary Material 1.

## Data Availability

The datasets used and/or analysed during the current study are available from the corresponding author on reasonable request.

## Abbreviations

PA: Physical activity
UGS: Urban green spaces
