# Supplementary material for: Barriers to youth physical activity in urban green spaces: evidence from a Turkish city
Source: BMC Public Health. 2026 Mar 10;26:1255. doi: 10.1186/s12889-026-26952-x (PMC13085498; doi:10.1186/s12889-026-26952-x)
Supplement: Supplementary file 1 — Supplementary Material 1. [file 12889_2026_26952_MOESM1_ESM.docx]

**Supplementary Material 1: Questionnaire**

**Dear Participant,**

You are invited to participate in a research study conducted by İbrahim Pehlivan entitled **“Identifying the Characteristics of Urban Green Spaces That Influence the Physical Activity Levels of Youth Living in Çankırı.”** Before deciding whether or not to participate, it is important that you understand why the research is being conducted and how it will be carried out.

Please read this form carefully and make sure you understand its contents. If you have any questions or need further clarification, feel free to ask the researcher directly at any time during the questionnaire.

- Participation in this study is entirely voluntary.
- You have the right to decline participation or to withdraw from the study at any time without any consequences.
- Please answer the questions freely, without any external pressure or influence.
- All personal data collected through this form will be kept strictly confidential and used solely for research purposes.
- Completing the questionnaire will be interpreted as your informed consent to participate in the study.

**QUESTIONNNAIRE**

This questionnaire is part of the master's thesis conducted by İbrahim Pehlivan at Çankırı Karatekin University, entitled “Identifying the Characteristics of Urban Green Spaces That Influence the Physical Activity Levels of Youth Living in Çankırı.”

**Section 1: Demographic Information**

1. **Age**
2. 15-19
3. 20-24
4. **Gender**
5. Female
6. Male
7. **Which neighborhood do you currently live in within Çankırı?**

(............................................................................................................)

**Section 2: Physical Activity Preferences**

1. **How many days per week do you engage in physical activity?**
2. Daily
3. ≥3 days per week
4. 2 days per week
5. 1 day per week
6. Less frequent
7. No physical activity
8. **Which types of urban green spaces do you use for physical activity?**
9. Residential green spaces
10. Nearby neighbourhood parks
11. Large public parks
12. **What kinds of physical activities do you regularly perform in urban green spaces?**

(Multiple answers can be selected)

A) Enjoying the scenery

B) Walking

C) Running

D) Walking the dog

E) Doing fitness exercises

F) Playing ball games

G) Cycling

H) Other (please specify)

1. **What types of facilities are available in the urban green spaces you frequently use for physical activity?**

(Multiple answers can be selected)

A) Walking paths

B) Bicycle lanes

C) Resting areas

D) Outdoor fitness equipment

E) Sports fields

F) Other (please specify)

1. **How long does it take you to reach the urban green space you frequently use for physical activity by walking?**
2. Less than 10 minutes
3. 15 minutes
4. 15–30 minutes

D) More than 30 minutes

**Section 3: Factors Affecting the Use of Urban Green Spaces**

1. **Which features of the urban green spaces you regularly use act as barriers to physical activity?** (Multiple answers can be selected)
2. Unattractive design
3. Low density of green areas
4. Lack of fitness equipment
5. Absence of open recreational spaces
6. Limited area size
7. Lack of water features
8. Inconvenient location
9. Limited plant diversity
10. Poor maintenance of vegetation
11. Lack of perceived safety
12. Overcrowding
13. Lack of parking facilities
14. Poor cleanliness
15. Lack of sports fields
16. Inadequate general upkeep
17. Lack of seating and resting facilities
18. Poor condition of the facilities
19. Lack of facilities suited to specific exercise interests
20. Presence of stray animals
21. Other (please specify)
22. **Which features of the urban green spaces you regularly use encourage physical activity?** (Multiple answers can be selected)
23. Sufficient size of the area
24. Availability of parking spaces
25. Rich vegetation
26. Absence of open recreational spaces
27. Presence of water surfaces
28. Well-maintained vegetation
29. Adequate general maintenance
30. Well-maintained facilities
31. Availability of resting areas
32. Sufficient fitness equipment
33. Adequate sports fields
34. Availability of open space
35. Convenient and accessible location
36. Not being crowded
37. Safety
38. Facilities suited to specific exercise interests
39. Absence of stray animals
40. Other (please specify)

**Section 4: Satisfaction with Urban Green Spaces**

1. **Please indicate your level of satisfaction with the UGS where you engage in physical activity**
2. Very dissatisfied
3. Dissatisfied
4. Neutral
5. Satisfied
6. Very satisfied

**Thank you for your participation.**
